# The effect and safety of monoclonal antibodies to calcitonin gene-related peptide and its receptor on migraine: a systematic review and meta-analysis

**DOI:** 10.1186/s10194-017-0750-1

**Published:** 2017-04-07

**Authors:** Min Hou, Haiyan Xing, Yongqing Cai, Bin Li, Xianfeng Wang, Pan Li, Xiaolin Hu, Jianhong Chen

**Affiliations:** 1 Department of Pharmacy, Institute of Surgery Research, Daping Hospital, Third Military Medical University, Chongqing, 400042 People’s Republic of China; 2 China Pharmacy Publishing House, Chongqing, 500000 People’s Republic of China

**Keywords:** Migraine, Monoclonal antibodies to calcitonin gene-related peptide, CGRP-mAb, Meta-analysis

## Abstract

**Background:**

Migraine has been recognized as one of the leading causes of disability in the 2013 Global Burden of Disease Study and seriously affects the quality of patients’ life, current treatment options are not ideal. Monoclonal antibodies to calcitonin gene-related peptide and its receptor (CGRP-mAbs) appear more promising for migraine because of considerably better effect and safety profiles. The objective of this study is to systematically assess the clinical efficacy and safety of CGRP-mAbs for migraine therapy.

**Methods:**

A systematic literature search in PubMed, Cochrane Library and Baidu Scholar was performed to identify randomized controlled trials (RCTs), which compared the effect and safety of CGRP-mAbs with placebo on migraine. Regarding the efficacy, the reduction of monthly migraine days from baseline to weeks 1–4, 5–8, and 9–12; responder rates were extracted as the outcome measures of the effects of CGRP-mAbs. Regarding the safety, total adverse events, the main adverse events, and other adverse events were evaluated.

**Results:**

We found significant reduction of monthly migraine days in CGRP-mAbs vs. placebo (weeks 1–4: SMD −0.49, 95% CI −0.61 to −0.36; weeks 5–8: SMD −0.43, 95% CI −0.56 to −0.30; weeks 9–12: SMD −0.37, 95% CI −0.49 to −0.24). 50% and 75% responder rates (OR 2.59, 95% CI 1.99 to 3.37; and OR 2.91, 95% CI 2.06 to 4.10) were significantly increased compared with placebo. There was no significant difference in total adverse events (OR 1.17, 95% CI 0.91 to 1.51), and the main adverse events including upper respiratory tract infection (OR 1.44, 95% CI 0.82 to 2.55), nasopharyngitis (OR 0.59, 95% CI 0.30 to 1.16), nausea (OR 0.61, 95% CI 0.29 to 1.32), injection-site pain (OR 1.73, 95% CI 0.95 to 3.16) and back pain (OR 0.97, 95% CI 0.49 to 1.90) were not obviously changed compared with placebo control, but the results showed significant increase of dizziness in CGRP-mAbs vs. placebo (OR 3.22, 95% CI 1.09 to 9.45).

**Conclusions:**

This meta-analysis suggests that CGRP-mAbs are effective in anti-migraine therapy with few adverse reactions, but more and larger sample-size RCTs are required to verify the current findings.

## Background

Migraine is a common, chronic neurovascular disorder with a female prevalence of 17% and a male prevalence of 9%, typically characterized by disabling attacks of severe headache and autonomic nervous system dysfunction [1, 2]. Different pathological and genetic mechanisms may be related to a variety of clinical manifestations. The etiology and pathogenesis of migraine are not yet completely understood.

Although studies on molecular players involved in the disease are incomplete, recent preclinical and clinical findings indicate that there is a clear correlation between migraine and the release of the neurotransmitters and vasoactive substances, such as 5-hydroxytryptamine (5-HT) [3–5], calcitonin gene-related peptide (CGRP) [6–8], and dopamine (DA) [9–11]. Over the past decades, ergotamine and the triptans, both of which are serotonin 5-HT agonists, have been proved effective for treating acute migraine, and are widely used in clinical practice [12, 13]. However, a significant number of migraine cases do not respond to these therapies. In addition, adverse effects such as cardiovascular concerns limit their use [14]. Calcitonin gene-related peptide (CGRP), is recognized as a crucial peptide in the pathophysiology of migraine, and has been increasingly investigated. CGRP receptor antagonists (CGRP-RAs), including olcegepant (BIBN4096BS), telcagepant, MK-3207 and MK-0974, have shown considerable efficacy in the treatment of migraine. However, several were discontinued due to concerns for hepatotoxicity with daily use [15–20], others were discontinued for other (or unknown) reasons.

Recently developed CGRP-mAbs were triggered much interest in the migraine community [21–24]. LY2951742 (Arteus Therapeutics), ALD403 (Alder Biopharmaceuticals), TEV-48125 (previously named as LBR-101) (Labrys Biologics-TEVA) and AMG 334 (Amgen) have completed Phase II and are undergoing III clinical trials [24]. These drugs have show anti-migraine activity with few adverse events. However, their clinical efficacy and safety lack systematic evaluation. Therefore, we performed a systematic review and meta-analysis on the overall efficacy and safety of CGRP-mAbs for migraine based on recent clinical findings.

## Methods

### Literature search and inclusion criteria

Two reviewers (MH and HYX) independently searched PubMed, Cochrane Library and Baidu Scholar for articles by entering “headache” or “migraine” and “monoclonal anti-CGRP antibody” or “monoclonal antibody to calcitonin gene-related peptide” as search terms. We then examined all articles and their reference lists to expand potentially relevant articles. The searches were limited to human studies published in English from inception of the databases to Nov 1, 2016. The articles were included in the meta-analysis if they met the following criteria: (1) randomized controlled trials (RCTs) evaluating the efficacy and safety of CGRP-mAbs for migraine; (2) no restrictions on population characteristics, blind and publication type; (3) participants diagnosed with migraine by using the recognized criteria, such as the International Classification of Headache Disorders, third edition (ICHD-3, beta version) and the International Classification of Headache Disorders (ICHD-II) [25–27] and (4) monotherapy with CGRP-mAbs and placebo in any form or in any dose or in any administration methods as treatment group and control group respectively. Studies were excluded when one of the following situations occurs: (1) subjects were animals; (2) interventions were drug combinations; and (3) except for RCTs, other types of trials such as cross-over designs, healthy controlled trials and self-contrast trials. Disagreement between two reviewers was settled by consensus or consultation with a third author (JHC or YQC).

### Quality assessment

The quality we studies was assessed independently by two investigators using the 7-item criteria in Review Manager Software version 5.3 provided by the Cochrane Collaboration [28]. The 7-item criteria mainly contained: (1) random sequence generation; (2) allocation concealment; (3) blinding of participants and personnel; (4) blinding of outcome assessment; (5) incomplete outcome data; (6) selective reporting and (7) other bias. Each item involved assigning a judgment of high, low, or unclear risk of material bias. Detailed criteria for making judgments about the risk of bias from each of the items in the tool are available in the Cochrane Handbook [29]. Discrepancies were reconciled by discussing with corresponding author.

### Data extractions and syntheses

The data with regard to the reduction of monthly migraine days was continuous, and presented as the mean ± SD for the meta-analysis. The expression of the mean ± SE was converted to the mean ± SD based on the principles of the Cochrane Handbook for Systematic Reviews of Interventions [30]. For the graphic, the original means and SDs were extracted with the aided software GetData Graph Digitizer 2.25 (http://getdata-graph-digitizer.com/) [31]. The data syntheses were performed by RevMan 5.3 (Cochrane Collaboration, Oxford, England). The reduction of monthly migraine days for each trial was analyzed by calculating standardized mean difference (SMD) with 95% confidence intervals (CI) with a fixed- or random- effect model. Other data including responder rates and adverse events were dichotomous, and were calculated by odds ratio (OR) with 95% confidence intervals (CI) with a fixed- or random-effect model.

### Heterogeneity analysis

The extent of heterogeneity may influence the results and conclusions. Therefore it is necessary to perform meta-analysis using Chi-square test. *I* ^*2*^ values smaller than 50% indicate no significant heterogeneity, and are acceptable. The fixed-effect model of analysis is then appropriate. Otherwise, the random-effect model is considered [32, 33].

### Risk of publication bias

Some situations may result in publication bias of the meta-analysis. On the one hand, studies with negative effects might not be published, and left out of the literature. On the other hand, studies without sufficient data might have been excluded based on the inclusion and exclusion criteria. Therefore, funnel plots were used to detect publication bias of the meta-analysis. When the funnel plot was approximately symmetrical and a majority of studies were located at its superior part, it was considered that there was no significant publication bias in the meta-analysis [31, 34].

## Results

### Study selection and inclusion

**Fig. 1:**
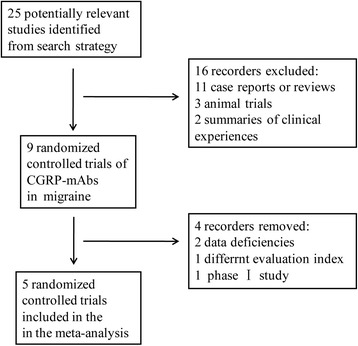
Process of identifying eligible studies for the meta-analysis

A total of five references were retrieved in our search [35–39]. Based on the title and abstract, 16 relevant articles were excluded due to their nature as case report or review, or summary of clinical experiences, or not being clinical trials (Fig. 1). Among five selected articles, two trials were TEV-48125 trials, and others were ALD403, AMG 334, and LY2951742. The characteristics of the five included studies were summarized in Table 1.

**Table 1:** Characteristics of the included studies

Included trials	Country	Study design	Eligibility criteria	Gender (male/female); mean age (years)		Migraine-days (hours) per month		Interventions drug	Course of treatment	Main efficacy outcomes	Most frequent adverse events
				Trial	Control	Trial	Control				
David W Dodick et al., 2014 (1) [37]	USA	RCT	ICHD-II 2004	19/88; 40.9 ± 11.4	14/96; 41.9 ± 11.7	6.7 ± 2.4	7.0 ± 2.5	LY2951742 (150 mg)	12 weeks	The reduction of monthly migraine days; 50%, 75%, and 100% responder rate	Injection site pain, nasopharyngitis, respiratory infection, nausea, and so on.
David W Dodick et al., 2014 (2) [38]	USA	RCT	ICHD-II 2004	14/67; 38.6 ± 10.8	16/66; 39.0 ± 9.6	8.4 ± 2.1	8.8 ± 2.7	ALD403 (1000 mg)	24 weeks	The reduction of monthly migraine days; 50%, 75%, and 100% responder rate; HIT-6; MSQ	Respiratory and urinary infection, back pain, nausea, and so on.
Hong Sun et al., 2016 [39]	USA	RCT	ICHD-II 2004	25/82; 42.6 ± 9.9	28/132; 41.4 ± 10.0	8.6 ± 2.5	8.8 ± 2.7	AMG 334 (7/21/70 mg)	12 weeks	The reduction of monthly migraine days; 50% responder rate; MIDAS; MSQ; HIT-6; PROMIS	Upper respiratory tract infection, back pain, nausea, and so on.
Marcelo E Bigal et al., 2015 (1) [35]	USA	RCT	ICHD-3 2013	15/82; 40.7 ± 12.6	12/92; 42.0 ± 11.6	80.4 ± 36.6 (hours)	82.1 ± 49.3 (hours)	TEV-48125 (225/675 mg)	12 weeks	The reduction of monthly migraine days; 50% and 75% responder rate	Upper respiratory tract infection, back pain, nausea, and so on.
Marcelo E Bigal et al., 2015 (2) [36]	USA	RCT	ICHD-3 2013	12/75; 41.5 ± 12.9	13/76; 40.7 ± 11.5	16.4 ± 5.3	16.8 ± 5.0	TEV-48125 (225/675/900 mg)	12 weeks	The reduction of monthly migraine days; 50% and 75% responder rate	Back pain, nasopharyngitis, injection-site pain, and so on.

*RCT* randomized controlled trial, *ICHD* the International Classification of Headache Disorders

### Quality of the included studies

**Fig. 2:**
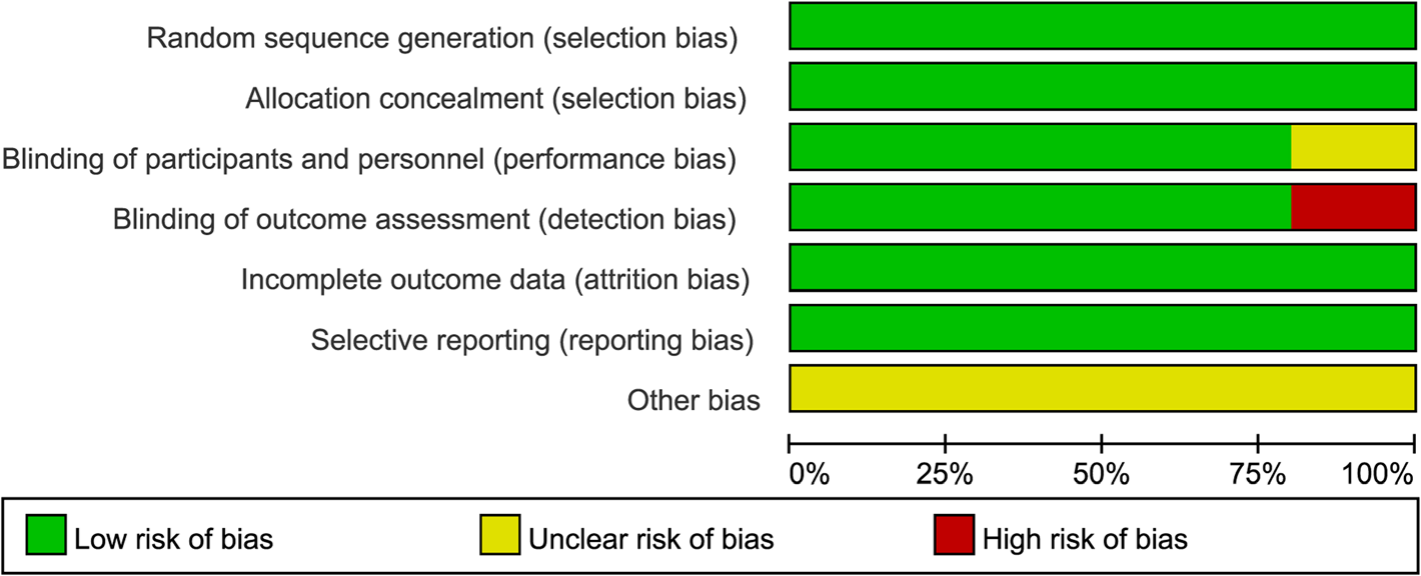
Risk of bias for included trials

**Fig. 3:**
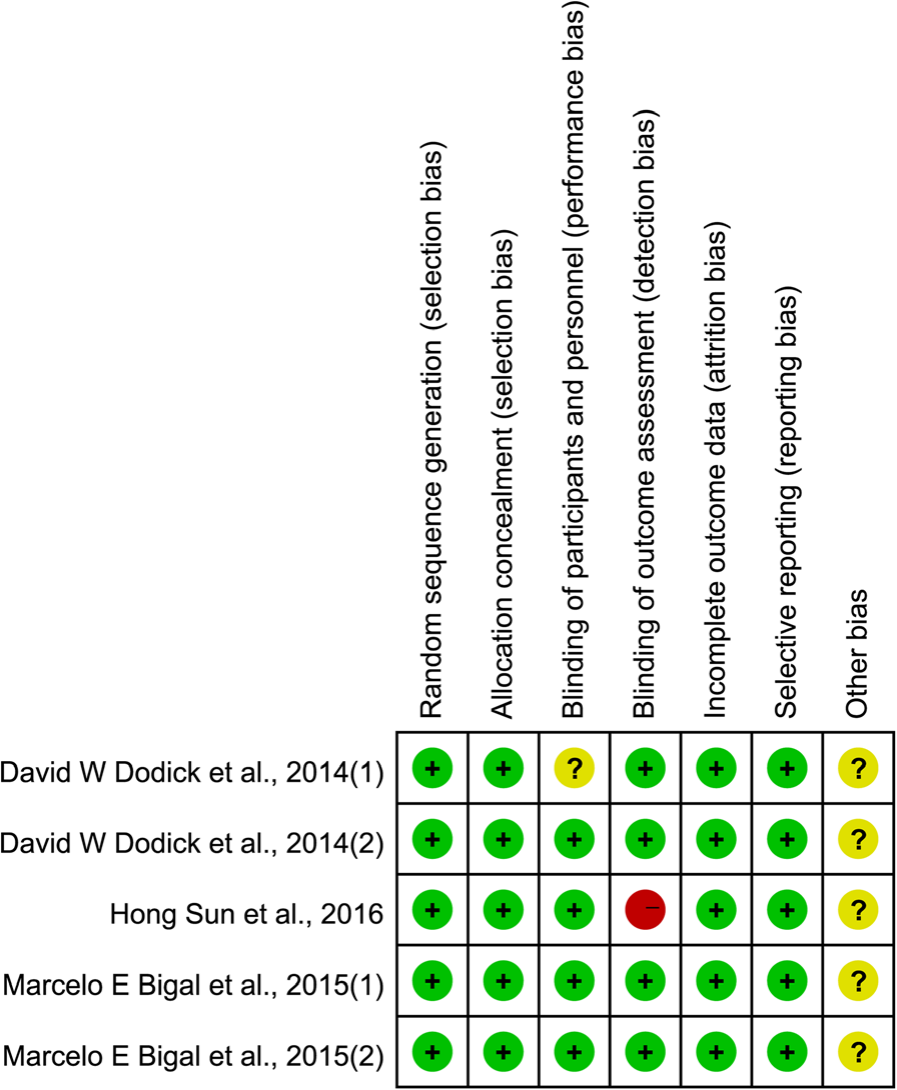
Risk of bias summery for included trials

The quality assessments of the included studies were summarized in Figs. 2 and 3. As listed in Figs. 2 and 3, all trials were randomized to receive CGRP-mAbs and placebo; participants from multicentres in the USA were randomly assigned by an interactive voice response or interactive web response system. Site investigators, patients, and sponsors were masked from treatment selection during the study. Only one study reported the pharmacists were aware of group assignment. But they had a sole responsibility was to drug accountability and preparation at each site [37]. Another reported the sponsored study personnel did not have access to patient-level clinical data apart from samples they were assaying and analysing [39] Overall, the included studies were suitable for the meta-analysis of the effect and safety of CGRP-mAbs for migraine.

## Effectiveness of CGRP-mAbs on migraine

### The reduction of monthly migraine days

**Fig. 4:**
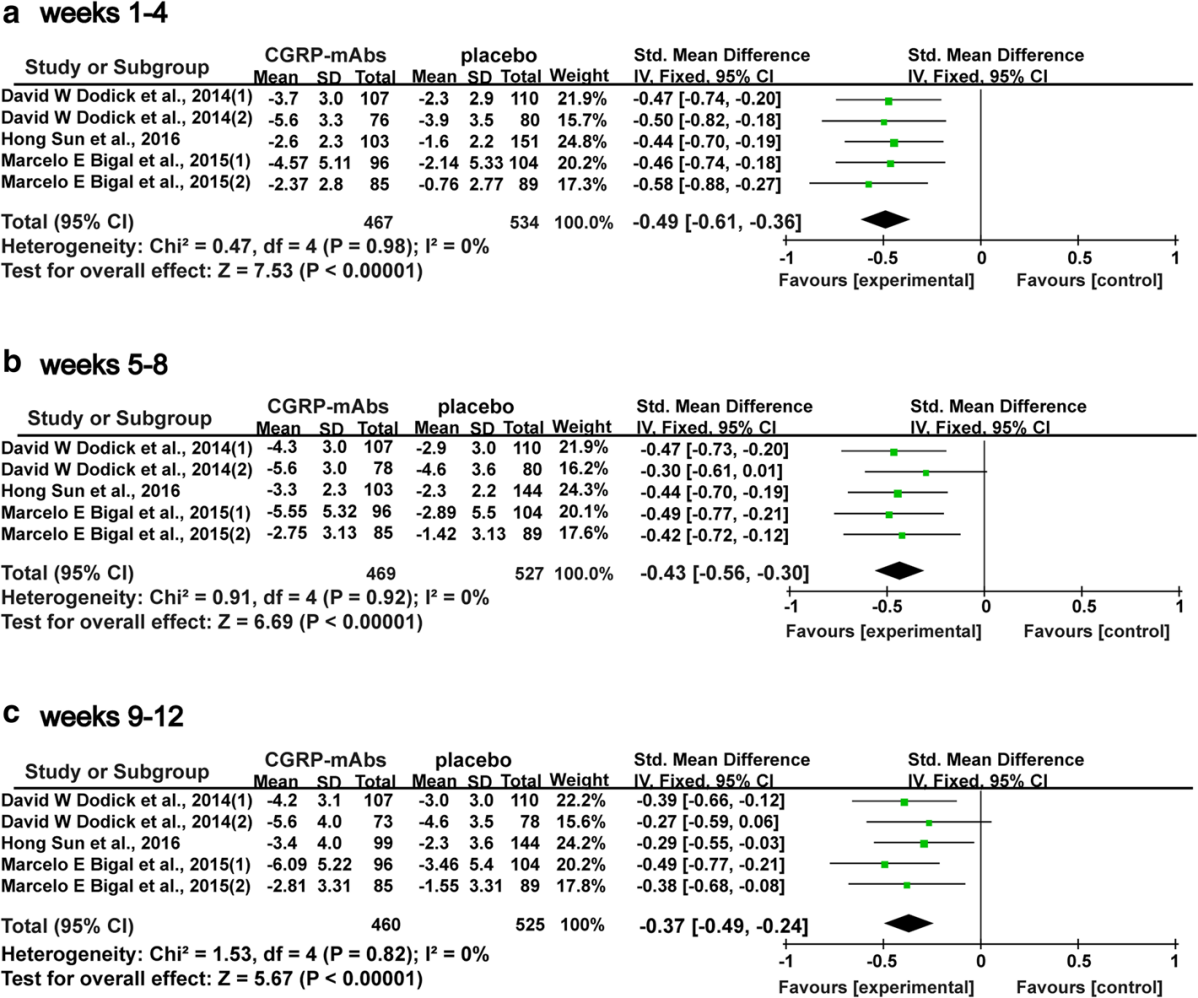
Forest plot of the meta-analysis showed a significant decrease in the numbers of monthly migraine days after therapy with CGRP-mAbs compared with placebo from baseline to weeks 1–4, 5–8, and 9–12. **a** The numbers of monthly migraine days of CGRP-mAbs and placebo at weeks 1–4; **b** The numbers of monthly migraine days of CGRP-mAbs and placebo at weeks 5–8; **c** The numbers of monthly migraine days of CGRP-mAbs and placebo at weeks 9–12. CGRP-mAbs, monoclonal antibodies to CGRP and its receptor; IV, inverse variance; CI, confidence interval

**Fig. 5:**
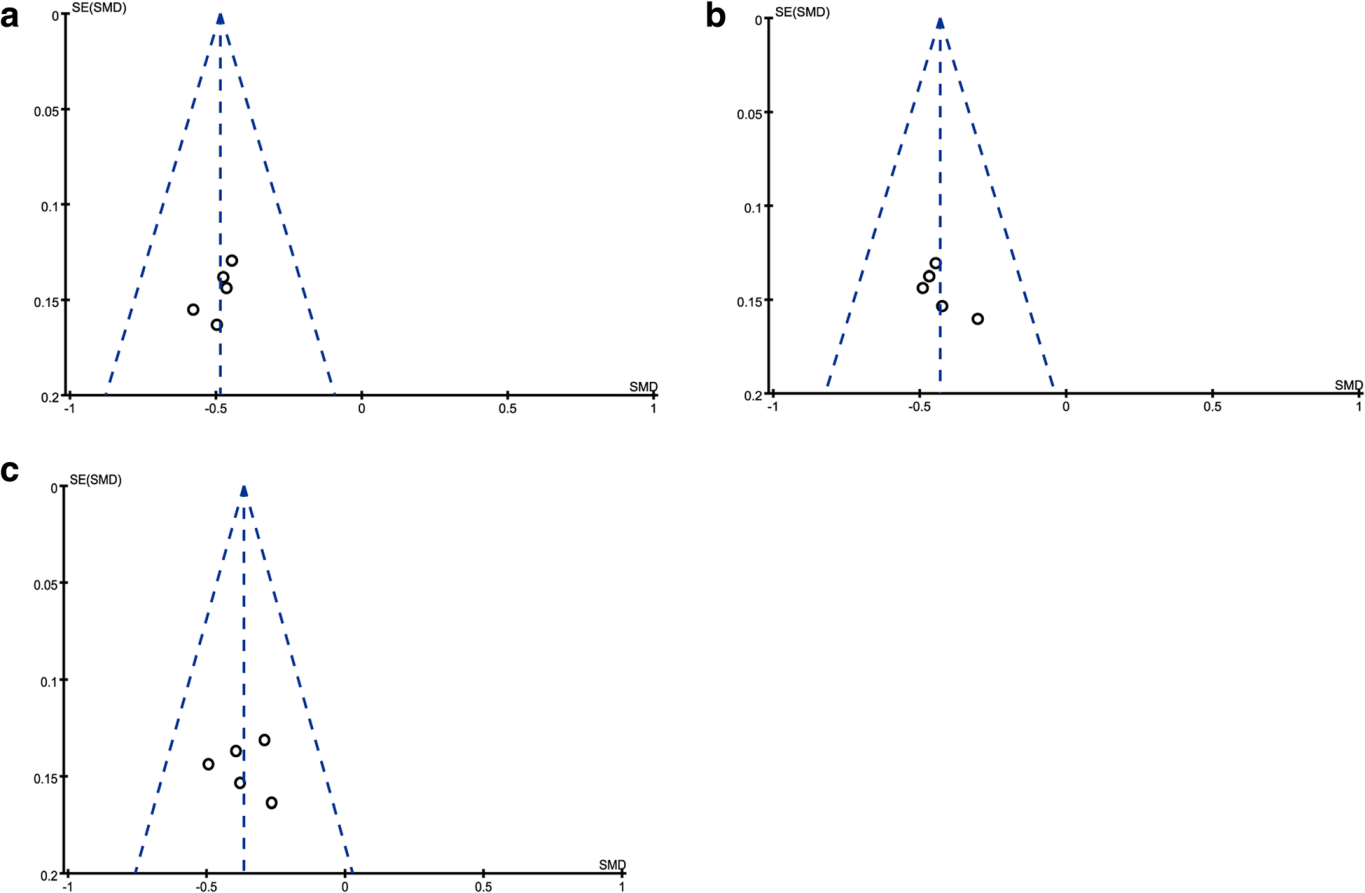
Funnel plot of the meta-analysis showed no significant publication bias in the reduction of monthly migraine days. **a** weeks 1–4; **b** weeks 5–8; **c** weeks 9–12. SE = Standard error, SMD = Standardized mean difference

All five trials (1001 subjects) included in this meta-analysis were evaluated for the change of monthly migraine days [35–39]. The data showed a significant decrease in the mean numbers of monthly migraine days after therapy with CGRP-mAbs compared with placebo, from a baseline to weeks 1–4, 5–8, and 9–12. As shown in Fig. 4, CGRP-mAbs showed a significant overall effect at weeks 1–4 (SMD −0.49, 95% CI −0.61 to −0.36) (Fig. 4a), weeks 5–8 (SMD −0.43, 95% CI −0.56 to −0.30) (Fig. 4b), and weeks 9–12 (SMD −0.37, 95% CI −0.49 to −0.24) (Fig. 4c). The *I* ^*2*^ value (*χ* ^2^ = 0.47/0.91/1.53, *P* = 0.98/0.92/0.82, *I* ^*2*^ = 0%) revealed a non-significant heterogeneity among the included trials. Risk of publication bias within studies was shown in Fig. 5. The inverse funnel plot was approximately symmetrical, thus there was no significant publication bias in the meta-analysis of the reduction of monthly migraine days.

### Fifty and seventy five percent responder rate

**Fig. 6:**
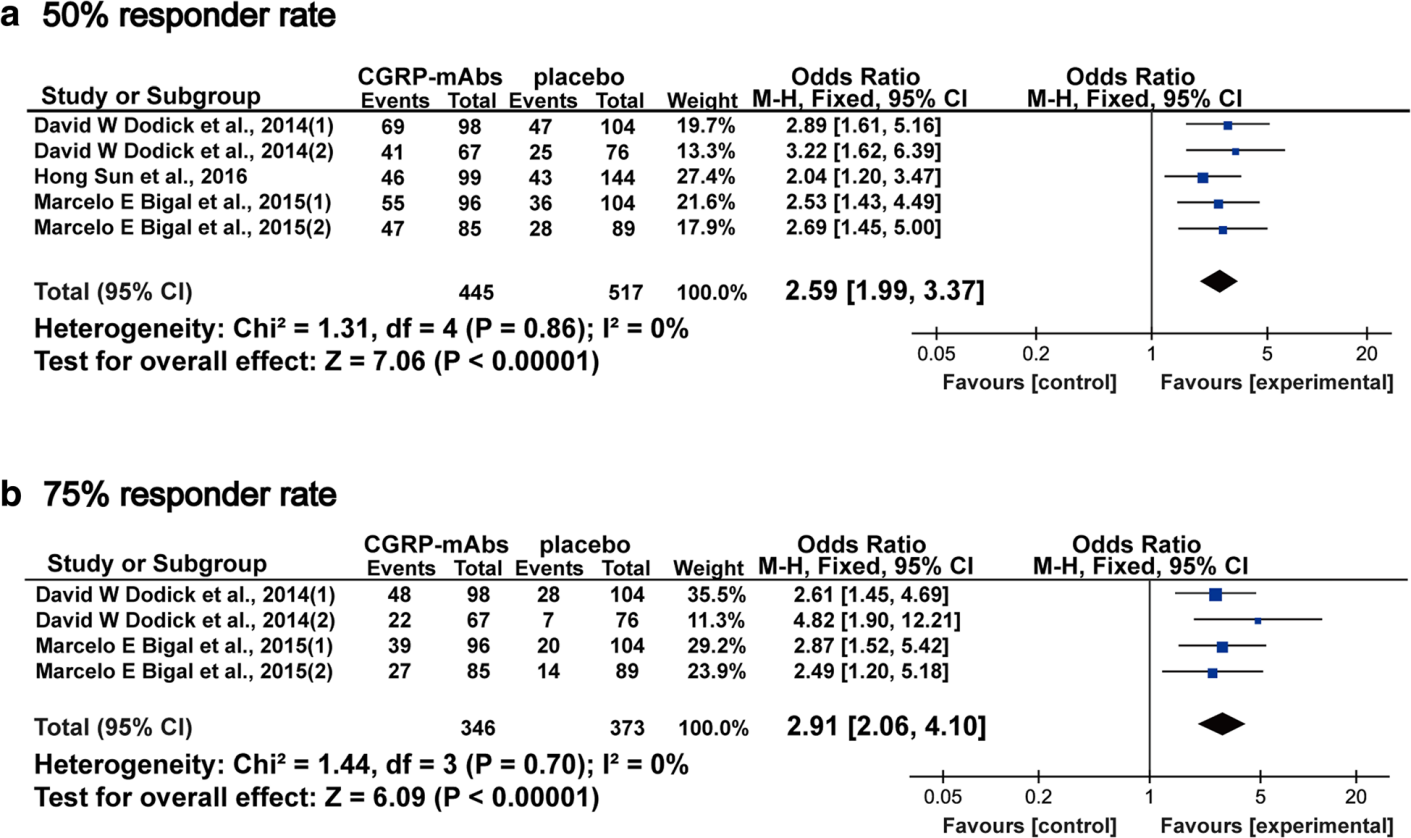
Forest plot of the meta-analysis showed significant decrease in 50 and 75% responder rates of CGRP-mAbs compared with placebo. **a** 50% responder rates; **b** 75% responder rates. CGRP-mAbs, monoclonal antibodies to CGRP and its receptor; M-H, Mantel-Haenszel; CI, confidence interval

**Fig. 7:**
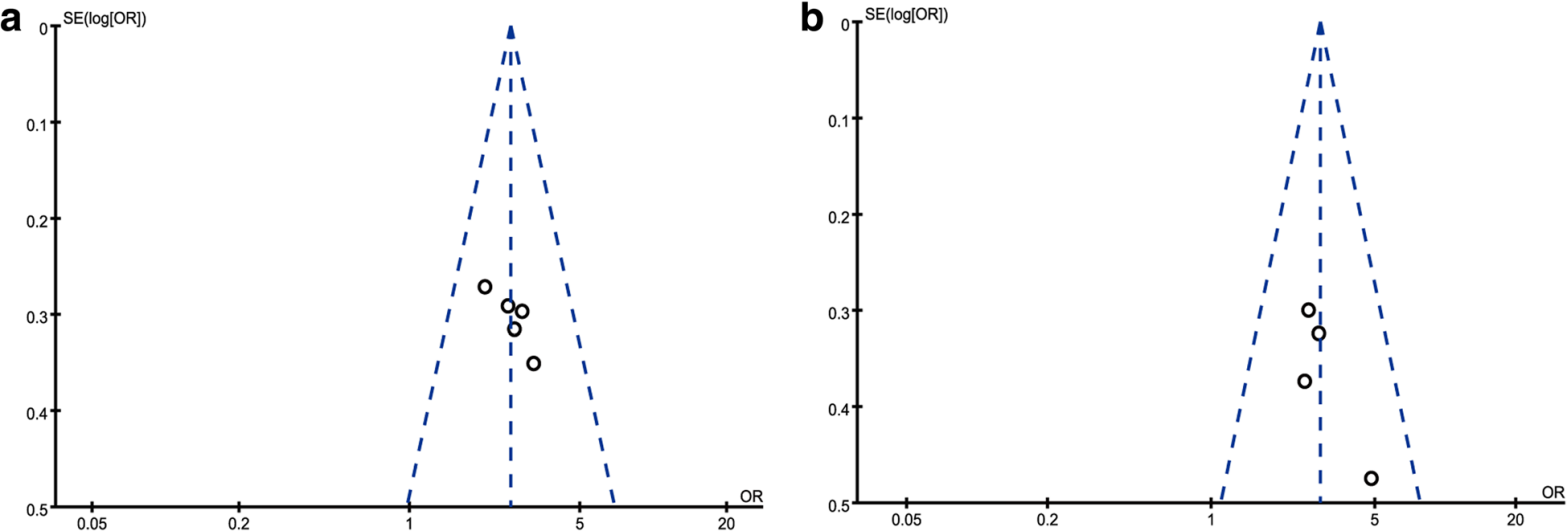
Funnel plot of the meta-analysis showed no significant publication bias in responder rate. **a** 50% responder rates; **b** 75% responder rates; SE = Standard error, OR = Odds Ratio

Fifty percent responder rate was counted in the five included studies with a total of 962 subjects [35–39], and 75% responder rate was counted in four included studies with a total of 719 subjects [35–38]. 100% responder rate was not analyzed due to the small sample size (only 2 trials), but the results with CGRP-mAbs were increased compared with placebo [37, 38]. Figure 6 showed significant decrease in 50 and 75% responder rates of CGRP-mAbs for migraine compared with placebo (OR 2.59, 95% CI 1.99 to 3.37; and OR 2.91, 95% CI 2.06 to 4.10). Furthermore, the meta-analysis results of the included tails found a low level of heterogeneity (*χ* ^2^ = 1.31 and 1.44, *P* = 0.86 and 0.70, *I* ^*2*^ = 0%). The funnel plot showed no obvious publication bias for the meta-analysis of responder rates (Fig. 7).

### Safety assessments of CGRP-mAbs for migraine

All five trials reported adverse events to different degrees. The frequent adverse events in patients receiving ALD403 were upper respiratory tract infection, urinary tract infection, fatigue, back pain, nausea and vomiting, and arthralgia [38]. Patients receiving LY2951742 reported upper respiratory infections and viral infections [37]. The common adverse events in migraine patients receiving TEV-48125 were mild injection-site pain, pruritus and erythema [35, 36]. The most frequently reported adverse events were nasopharyngitis, fatigue, and headache in migraine receiving AMG334 [39].

### Total adverse events

**Fig. 8:**
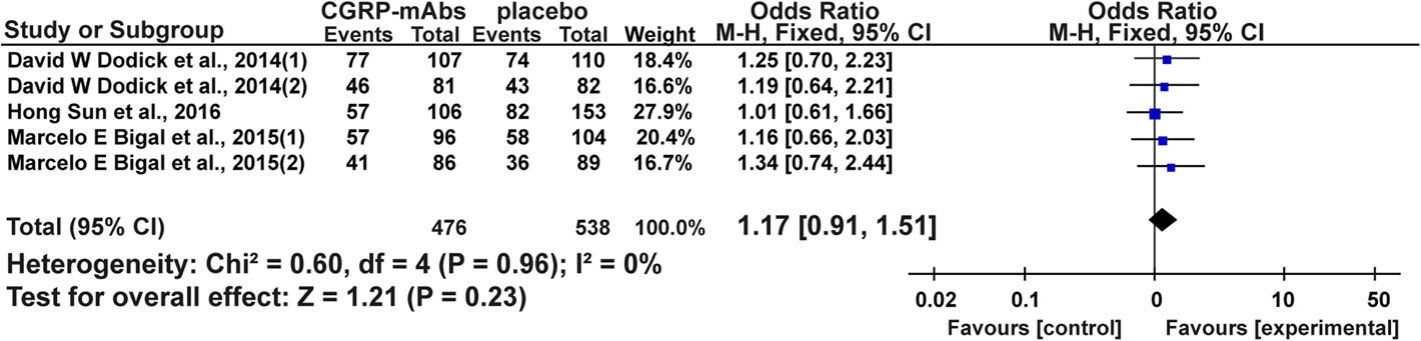
Forest plot of the meta-analysis showed non-significant difference in total adverse events of CGRP-mAbs compared with placebo. CGRP-mAbs, monoclonal antibodies to CGRP and its receptor; M-H, Mantel-Haenszel; CI, confidence interval

**Fig. 9:**
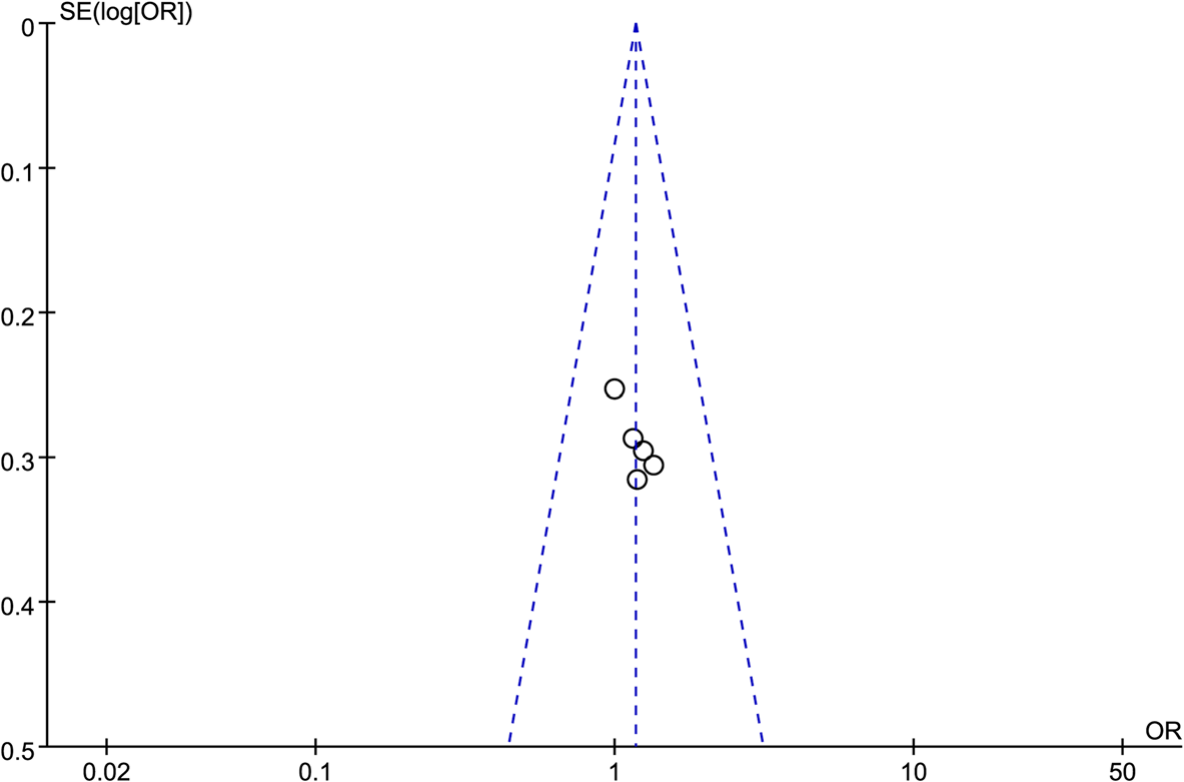
Funnel plot of the meta-analysis showed no significant publication bias in total adverse events. SE = Standard error, OR = Odds Ratio

All five studies reported a total of 571 patients with adverse events [35–39]. The meta-analysis results showed total adverse events in migraine patient with CGRP-mAbs therapy were not significantly different from those observed in placebo groups (OR 1.17, 95% CI 0.91 to 1.51) (Fig. 8). The findings suggest the safety of CGRP-mAbs for migraine. The results of meta-analysis showed that the included studies were highly homogeneous (*χ* ^2^ = 0.60, *P* = 0.96, *I* ^*2*^ = 0%). No obvious publication bias was found in the funnel plot (Fig. 9).

### Main adverse events

**Fig. 10:**
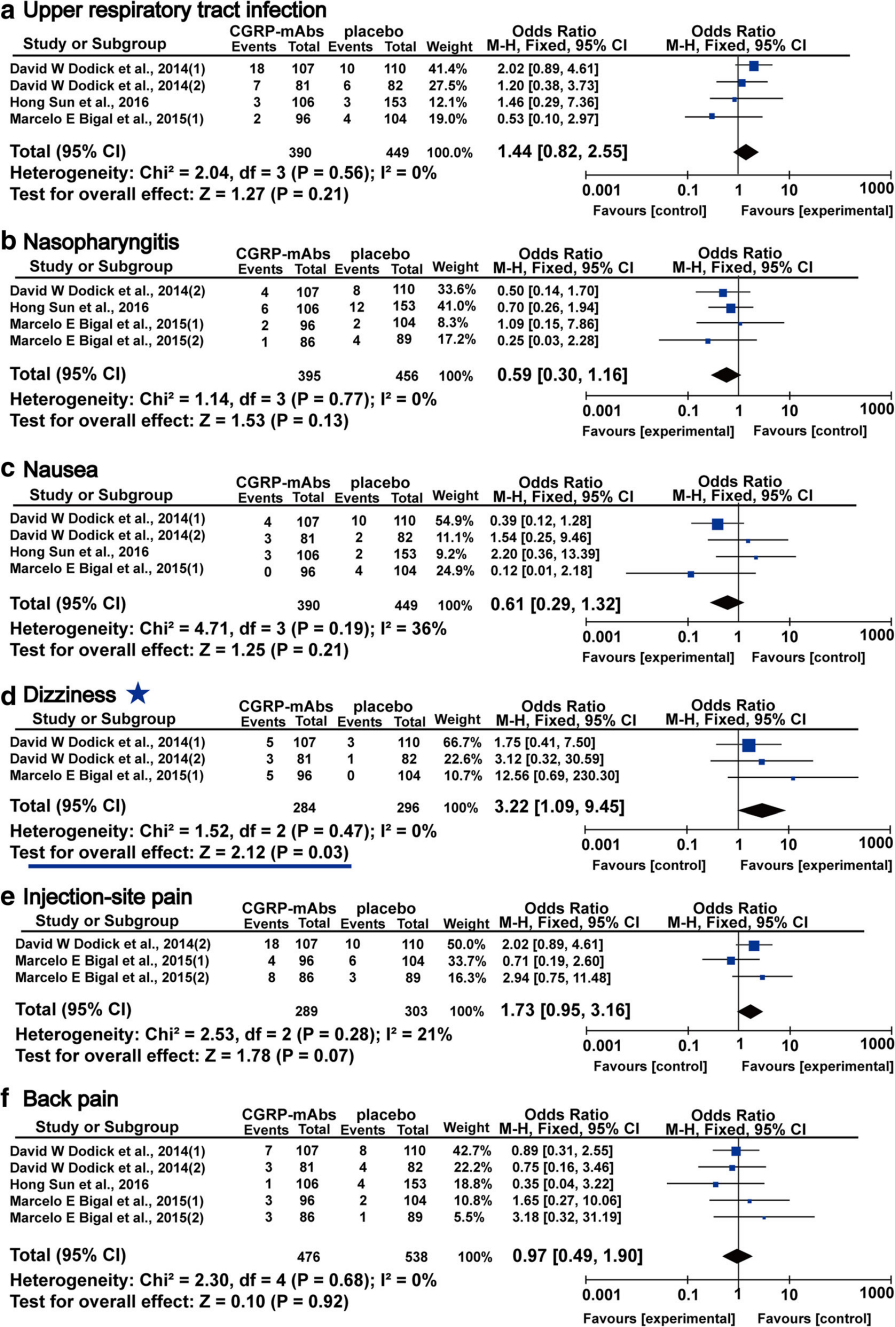
Forest plot of the meta-analysis showed non-significant difference in main adverse events of CGRP-mAbs compared with placebo. upper (**a**), respiratory tract infection; (**b**), nasopharyngitis; (**c**), nausea; (**d**), dizziness; (**e**), injection-site pain; (**f**), back pain. CGRP-mAbs, monoclonal antibodies to CGRP and its receptor; M-H, Mantel-Haenszel; CI, confidence interval. ★ The significant result was labeled with an asterisk

The most frequent adverse events in migraine with CGRP-mAbs were upper respiratory tract infection, nasopharyngitis, nausea, dizziness, injection-site pain and back pain [35–39]. As shown in Fig. 10, there was no obvious difference between CGRP-mAbs and placebo group in main adverse events (upper respiratory tract infection: OR 1.44, 95% CI 0.82 to 2.55 (Fig. 10a); nasopharyngitis: OR 0.59, 95% CI 0.30 to 1.16 (Fig. 10b); nausea: OR 0.61, 95% CI 0.29 to 1.32 (Fig. 10c); injection-site pain: OR 1.73, 95% CI 0.95 to 3.16 (Fig. 10e); back pain: OR 0.97, 95% CI 0.49 to 1.90 (Fig. 10f), but the significant increase of dizziness in CGRP-mAbs was found (OR 3.22, 95% CI 1.09 to 9.45) (Fig. 10d). All *I* ^*2*^ value revealed a non-significant heterogeneity among the included studies. The funnel plot was not created for the main adverse events due to the small sample size.

### Other adverse events

Other adverse events in migraine treated with CGRP-mAbs, such as sinusitis [35], headache [36, 39], and arthralgia [37], were reported in included studies. However, we did not conduct systematic analysis due to fewer number samples. Furthermore, there was no evidence of any effect of CGRP-mAbs on hepatotoxicity, only four patients had transient increases in liver enzyme concentrations during the treatment phase, which was considered non-treatment related [36]. No clinically important changes in cardio-cerebrovascular system or electrocardiograms (ECGs) or other vital signs were recorded.

## Discussion

### The role of CGRP in migraine

CGRP, a neuropeptide released from activated trigeminal sensory nerves, may play an important role in the pathophysiology of migraine. In preclinical studies, concentrations of CGRP in trigeminal ganglion (TG), trigeminal nucleus caudalis (TNC) and venous blood during migraine onset are elevated in rat or cat models compared with control [40–42]. Furthermore, relief of migraine pain coincides with reduction or normalization of CGRP concentrations in brain tissue and blood after treatment with 5-HT1B/1D or CGRP receptor agonists or also Botulinum toxin (BTX) [43, 44]. In clinical studies, serum and plasma levels of CGRP are increased in patients with migraine onsets as compared with healthy subjects [45–47]. In preclinical results, regulating disordered CGRP production may reduce continued occurrence of migraine after taking anti-migraine drug [48–51]. Based on these studies, it is possible to prevent migraine by blocking CGRP binding to its receptor.

### Strengths of CGRP-mAbs for treating migraine

In recent years, CGRP-RAs have been considered as a novel, approach to treating migraine because they showed promising efficacy in several clinical trials [8, 18–20, 52–54]. However, liver toxicity with their daily administration was found. Thus further developments of some CGRP-RAs including telcagepant MK-0974 and MK-3207 were terminated [18–20, 55], two CGRP-RAs, B144370A and BMS-927711, showed a good clinical efficacy in the phase II studies but their development status is not clear and it is not known whether these drugs showed liver toxicity [52, 54], and ubrogepant MK-1602, has been tested in two phase II trials, but the results have not been reported yet [55]. CGRP-mAbs are macromolecules made of proteins that directly target CGRP or its receptor. The mAbs are behaved to bind and neutralize the excessive CGRP release by perivascular trigeminal sensory nerve fibers. Clinical studies showed a remarkable effect on migraine with no abnormal liver side effect for in subject receiving CGRP-mAbs [24]. The satisfactory safety could be mostly because of its high target specificity with minimum off-target toxicity. Furthermore, as summarized in the five included article in this study [35–39], CGRP-mAbs were administered subcutaneously or intravenously, allowing for monthly or even quarterly dosing due to their long half-lives and absence of liver toxicity. These strengths should enhance the long-term usage of this therapy compared with oral drugs, which had to be taken once or twice daily. Meanwhile, it may avoid hepatotoxicity and drug-drug interactions, and become ideal candidates for preventive treatment of migraine. In the next years the potential advantages of CGRP-mAbs should also be evaluated in direct comparative studies with the available preventive treatments.

CGRP-mAbs are behaved to long half-lives for migraine prevention, and not cross the blood-brain barrier (BBB) due to a large particle size. The mechanisms through which CGRP relieves migraine and the precise site of action of CGRP-mAbs are not completely understood. CGRP is expressed both centrally and peripherally. Recently, the view that there is no clear proof of breakdown or leakage in the BBB during migraine attack has emerged [56–58], so macromolecular drugs may not cross the BBB. Another view is that these macromolecular drugs act mostly peripherally for prophylaxis [55, 59, 60]. However, these hypotheses have not been verified. In the next years the potential mechanisms of CGRP-mAbs should be explored by pharmacological methods.

### Clinical efficacy and safety of CGRP-mAbs for migraine in the meta-analysis

This meta-analysis examined the efficacy and safety of CGRP-mAbs in comparison with placebo for the treatment of migraine, and validated CGRP as a therapeutic target. In our study, five randomized controlled trials involving 1001 participants were included. The results indicated CGRP-mAbs as effective in migraine prevention. Furthermore, safety data from the five trials involving 1014 participants suggest that the main adverse events including upper respiratory tract infection, nasopharyngitis, nausea, injection-site pain, back pain and other adverse events were not different between treatment and placebo group except for dizziness. Thus, CGRP-mAbs had a favorable safety profile, and there were no specific adverse events as seen in phaseI study [61, 62]. The possibility of dizziness related to chronic depletion of systemic levels of CGRP still needs to be evaluated in long-term treatment studies. This meta-analysis supports the importance of CGRP in the pathophysiology of migraine.

### Limitations of the meta-analysis

This meta-analysis also has some limitations. First, numerous phase III trials of CGRP-mAbs are undergoing without conclusion and publication so far [24, 55]. Only five studies were included in our analysis. Second, some were completed by three lead authors between the five studies [35–38], which may contribute to publication bias. Third, the evaluation indexes are relatively simple, and may have affected our meta-analysis results. Especially the efficacy of CGRP-mAbs for migraine, only two evaluation indexes, including reduction of monthly migraine days and responder rates, were included. In order to comprehensively evaluate the effects of CGRP-mAbs, quality of life in migraine should be assessed using the Role Function-Restrictive (RFR), Role Function-Preventive (RFP), and Emotional Function (EF) subscales of the Migraine-Specific Quality of Life Questionnaire (MSQ), Headache Impact Test (HIT-6), Migraine Disability Assessment (MIDAS), and Patient-reported Outcomes Measurement Information System (PROMIS). Nevertheless, considering that only two studies [37, 39] conducted the relative indexes, meta-analysis was not performed. Furthermore, there was no long-term follow-up of the participants, so longer term safety and efficacy of CGRP-mAbs remains unknown.

## Conclusions

In conclusion, based on the results of 5 Phase II trials, this review and meta-analysis revealed a significant effect of CGRP-mAbs for migraine prevention with few adverse reactions. Ongoing Phase III multicenter RCTs will need to be analyzed for publication for whether they reproduce these findings. Furthermore, given that RCTs are designed to test a therapeutic hypothesis under an optimal setting, several factors may comprise their strict and controlled conditions and thus restrict their application to real-world clinical practice, the mathematical views provided by RCTs should be tested in real-life studies that confirmed the positive result of previous clinical trials, and weighed against the flexibility of clinical practice and real-world settings in the future.

## Abbreviations

5-HT: 5-hydroxytryptamine
BBB: The blood-brain barrier
CGRP: Calcitonin gene-related peptide
CGRP-mAb: Monoclonal antibodies to calcitonin gene-related peptide
CI: Confidence interval
DA: Dopamine
ICHD: The international classification of headache disorders
IV: Inverse variance
M-H: Mantel-Haenszel
RCT: Randomized controlled trial
